# Elucidating the effect of body mass index, height, and parity on uncomplicated cystitis: a nationwide population-based cohort study

**DOI:** 10.1038/s41598-022-06425-y

**Published:** 2022-02-11

**Authors:** Filip Jansåker, Xinjun Li, Kristina Sundquist

**Affiliations:** 1 Center for Primary Health Care Research, Department of Clinical Sciences Malmö, Clinical Research Centre (CRC), Skåne University Hospital, Lund University, Jan Waldenströms gata 35, 205 02 Malmö, Sweden; 2 Department of Family Medicine and Community Health, Department of Population Health Science and Policy, Icahn School of Medicine at Mount Sinai, New York, USA; 3 Center for Community-Based Healthcare Research and Education (CoHRE), Department of Functional Pathology, School of Medicine, Shimane University, Matsue, Japan

**Keywords:** Urogenital diseases, Risk factors, Bacterial infection, Epidemiology, Epidemiology

## Abstract

In this nationwide cohort of one million fertile women, BMI, height, and parity only had minor but statistically significant effects on the risk of uncomplicated cystitis. The results indicate that underweight women and certain sociodemographic groups might have higher risks, which could have underlying explanations that need further studying.

## Introduction

Cystitis is a very common urinary tract infection (UTI) in otherwise healthy fertile women^1^. The distance uropathogens must travel from the fecal reservoir to the urethra has previously been found to be related to cystitis^2,3^ and associations with height and body mass index (BMI) have been found^2^. Although underweight and obesity have been associated with various infections^4,5^, only a few smaller studies have investigated BMI in relation to UTI with inconsistent results^3,5–8^ and the effect of height on cystitis does not seem to be well studied. By using nationwide primary healthcare registers of high quality, we aimed to elucidate if BMI, height, and other factors affect the risk of cystitis in fertile women.

### Methodology

This open cohort study consisted of 1,073,467 fertile females aged 15–50 years during the study time period (1997–2018). The main predictor variables were BMI, height, and parity. Each woman was included once.

### Predictor variables and confounders

*BMI* and *Height*: Continuously and categorically with BMI < 18.5 (underweight); BMI 18.5–24.9 (normal weight); BMI 25.0–29.9 (overweight); and BMI ≥ 30.0 (obesity). *Parity*: continuous. Confounders were *sociodemographic factors*^9,10^.

### Outcome criteria

The outcome was defined as the first event of an acute uncomplicated cystitis (in this manuscript called cystitis) during the study time period. Nationwide primary healthcare data were used to identify the outcome cystitis, defined as “N30” according to the *10th revision of the International Classification of Diseases.* The study did not include subtypes of cystitis that were not classified as acute infective cystitis (i.e., N301-4 and N308), nor women with comorbidities or drug therapy not aligned with an uncomplicated infection: e.g., immunodeficiency disorder, diabetes mellitus, urological abnormalities, or redeemed prescription on anti-neoplastic and/or immunomodulating agents^9,10^.

### Data sources and sampling

Considering that the vast majority of all cystitis occur in primary healthcare settings, the study used primary healthcare data to identify the study population and the outcome^9^. The Swedish Medical Birth Register^11^ was used to identify the main predictor variables. The Total Population Register was used to collect data on emigration and sociodemographic factors. Other validated nationwide registers^12^ included the Outpatient-, Inpatient-, Cause of Death-, and Medical Prescription Registers. Data were linked using pseudonymized versions of the unique 10-digit identification number. A total of 2,052,873 women aged 15–50 years were identified during the study period, of these 1,073,467 women had data on parity, height, and BMI data and were included in the study population for the analysis; of these, 332,286 had an event of cystitis during the study period.

### Statistics

Descriptive statistics and incidence rates (IR) were calculated for each predictor variable. The study period started on January 1, 1997. Baseline was defined when a participant (≥ 15 years) was identified in the nationwide primary health data. Person‐years were calculated until outcome event, death, emigration, ≥ 50 years of age, or end of the study period (December 31, 2018). Cox regression models were used to estimate Hazard ratios (HR) and 95% confidence intervals (CI). Model 1: univariate model for each variable; Model 2: adjusted for height and BMI; Model 3: age and parity added in the adjustments; Model 4: fully adjusted. Only recorded fertile (parity ≥ 1) women with BMI and height data available were included. P-value < 0.05 was used to define statistical significance. SAS version 9.4 was used.

### Ethical considerations

The present study was a non-intervention nationwide register study based on pseudonymized secondary data obtained from the Swedish authorities and was approved by the Ethical Review Board in Lund (Sweden). The permission to take informed consent was formally waived. All methods were performed in accordance with the relevant guidelines and regulations.

## Results

The characteristics of the study population (N = 1,073,467) and distribution of cases (n = 332,286) are shown in Supplementary Table S1. The overall IR was 3.2 (95% CI 3.19–3.21) per 100 person-years (Supplementary Table S2). Table 1 demonstrates an inverse risk of cystitis associated with high BMI compared to normal BMI. On the other hand, the HR was somewhat higher in underweight women, i.e., 1.12 (95% CI 1.10–1.14), which remained higher in all models compared to the reference group. In the univariate and height-adjusted model, obesity was not associated with cystitis, but when including age, parity, and, furthermore, sociodemographic factors both obesity and overweight were to a minor degree inversely associated with cystitis. An increase in BMI (continuous) was inversely associated with cystitis: HR = 0.99 (95% CI 0.99–0.99; p < 0.0001) in Model 3–4 in Table S3. No clear association between height and cystitis was found, but a slightly higher and statistically significant risk of cystitis was observed in the two tallest groups of women. Young age, low education, or African, MENA, and Latin American/Caribbean origin were independently associated with higher risk of cystitis compared to their corresponding reference in the fully adjusted models. In Supplementary Table S4, the univariate model including all co-variates is shown. The associations between the main predictors (i.e., BMI, height, and parity) and cystitis were more or less similar in this model compared to the fully adjusted models (Tables 1 and S3), while the HR of cystitis associated with the sociodemographic factors were generally higher in the univariate model compared to the fully adjusted models.

**Table 1:** Inverse association between BMI and cystitis in fertile women (adjusted for height, parity, and sociodemographic factors).

Covariates	Model 1				Model 2				Model 3				Model 4			
	HR	95% CI		p-value	HR	95% CI		p-value	HR	95% CI		p-value	HR	95% CI		p-value
**Body mass index (BMI) (ref. 18.5–24.9)**																
< 18.5	1.12	1.10	1.14	< 0.0001	1.12	1.10	1.14	< 0.0001	1.14	1.12	1.16	< 0.0001	1.11	1.09	1.13	< 0.0001
25.0**–**29.9	0.98	0.97	0.99	< 0.0001	0.98	0.97	0.99	< 0.0001	0.95	0.94	0.96	< 0.0001	0.94	0.93	0.95	< 0.0001
≥ 30.0	1.00	0.98	1.01	0.6069	1.00	0.98	1.01	0.5251	0.94	0.93	0.95	< 0.0001	0.93	0.92	0.94	< 0.0001
**Body height (cm)**					1.00	1.00	1.00	0.0034	1.00	1.00	1.00	< 0.0001	1.00	1.00	1.00	< 0.0001
**Parity**									1.01	1.01	1.02	< 0.0001	1.01	1.00	1.01	0.0002
**Age (ref. age 45–50 years)**																
15**–**24									1.54	1.52	1.56	< 0.0001	1.47	1.45	1.49	< 0.0001
25**–**34									1.10	1.08	1.11	< 0.0001	1.07	1.06	1.09	< 0.0001
35**–**44									1.00	0.98	1.01	0.7112	0.97	0.96	0.99	0.0002
**Educational level (ref. ≥ 12 years)**																
≤ 9													1.18	1.16	1.19	< 0.0001
10**–**11													1.11	1.10	1.12	< 0.0001
**Family income (ref. high)**																
Low													1.07	1.06	1.08	< 0.0001
Middle low													1.05	1.04	1.06	< 0.0001
Middle high													1.03	1.02	1.04	< 0.0001
**Region of residence (ref. large cities)**																
Southern Sweden													0.93	0.92	0.93	< 0.0001
Northern Sweden													0.77	0.76	0.78	< 0.0001
**Country of origin (ref. born in Sweden)**																
Eastern Europe													1.04	1.02	1.05	< 0.0001
Western countries													1.04	1.02	1.06	< 0.0001
Middle East/North Africa													1.28	1.26	1.30	< 0.0001
Africa (excluding North Africa)													1.12	1.09	1.15	< 0.0001
Asia (excluding Middle East) and Oceania													0.98	0.96	1.00	0.0288
Latin America and the Caribbean													1.29	1.26	1.33	< 0.0001

Model 1: Univariate model; Model 2: Adjusted for body height; Model 3: Adjusted for body height, age, and parity; Model 4. Fully adjusted.

In Supplementary Figs. S1 and S2, the IRs of cystitis were plotted by BMI and country of origin and by BMI and age. Most groups had the highest IRs in underweight women. However, for some groups the IRs amplified in obese women, especially in women from Africa (excluding Northern Africa). For young women a clear decline in IR seemed to occur as BMI increased. For the other age groups, BMI seemed to be of less importance.

## Discussion

In this nationwide cohort of over one million women aged 15–50 years, BMI, height, and parity only had a minor impact on the risk of cystitis. However, as the results indicate, women who are underweight and of certain sociodemographic groups might be suffering disproportionately from this infection. Obesity and short height on the other hand seemed to be slightly protective against cystitis, where the tallest women had a slightly higher risk of cystitis. Although women who had given birth seemed to have an increased risk of cystitis in general compared to nullipara women^9,10^, an increase in parity did not seem to affect the risk of cystitis to a high extent.

To the best of our knowledge, this is the first nationwide study to elucidate the effect of BMI and height on cystitis. Previous studies, of different designs, have included some hundred to a couple of thousand cases and the results have been inconclusive^3,6–8^. Larger studies on other infections have, however, found similar results to ours. For example, while being underweight is associated with increased risk of other infections (e.g., pneumonia and influenza) the associations with overweight have been less clear^4,5^. Obesity has^13^ recently been associated with decreased mortality in septic patients^13^ but although no obvious mechanisms seem to underly this association, the authors elaborated that factors such as energy reserves and immune system activity related to obesity could be at play. However, for the potential effect of BMI on cystitis this seems to be less likely. Instead, an anatomical causality might be present, as BMI has been associated with the anogenital distance potential fecal uropathogens need to travel^2,3^ and it is possible that an increased abundance in subcutaneous tissue related to increased BMI might increase the anogenital distance. This could be further supported by the slightly inverse risk (HR 0.99) of cystitis with an increase in BMI (Supplementary Table S3). On the other hand, height have been associated with the distance uropathogens need to travel (p = 0.002)^2^ to a lesser extent, which could explain why height only seemed to have a weak, although statistically significant, association with cystitis in our study (Table 1).

Important additional findings are that the sociodemographic factors associated with UTI identified in two previous studies of ours^9,10^ remained. This consistency strengthens that there is an association between sociodemographic factors and UTI in general. The present study, however, also found a significantly increased risk (9–15%) in women from Africa not previously observed for cystitis^9^, and somewhat increased HRs for other groups of foreign-born women. This could be explained by the varying effect of BMI on cystitis in this category (Supplementary Fig. S1).

Major limitations are that this study did not have access to microbiological data or information on the clinical presentation leading to a diagnosis. Considering the large dataset extending over two decades these potential limitations were likely balanced by the strengths and non-differential. Major strengths were that the study involved several validated nationwide data registries^11,12^, as well as nationwide data from primary healthcare, which is quite unique compared to previous studies.

That young underweight women could be suffering disproportionately from cystitis is of concern as these underweight women already seem to be at increased risk of severe infections^4,5^ as well as morbidity and mortality in general^14^. Clinical implications of our findings are likely of minor concern for individual patients, but healthcare professionals could be aware of the increased risks of cystitis in certain patient groups.

In conclusion, the effect of BMI, height, and parity seemed to be of statistically significant but likely minor importance for cystitis in this nationwide study of fertile women. The findings of increased risks of cystitis associated with low BMI and among certain sociodemographic groups are of importance but more research is needed to identify the mechanisms behind the associations identified in this study.

## Supplementary Information

Supplementary Information.

## Data Availability

This study made use of several national registers and, owing to ethical and legal concerns, data cannot be made openly available. Further information regarding the health registries is available from the Swedish National Board of Health and Welfare: https://www.socialstyrelsen.se/en/statistics-and-data/registers/. The code used in the analysis can be provided upon request.

## Abbreviations

ATC: Anatomic therapeutic chemical classification system
BMI: Body-Mass-Index
CI: Confidence interval
HR: Hazard ratio
ICD: International classification of diseases
MENA: Middle East/North Africa
UTI: Urinary tract infection
