# Supplementary material for: Elucidating the effect of body mass index, height, and parity on uncomplicated cystitis: a nationwide population-based cohort study
Source: Sci Rep. 2022 Feb 11;12:2380. doi: 10.1038/s41598-022-06425-y (PMC8837615; doi:10.1038/s41598-022-06425-y)
Supplement: Supplementary file 1 — Supplementary Information. [file 41598_2022_6425_MOESM1_ESM.docx]

| **Supplementary Table S1**. The characteristics of the study population and cases of cystitis in fertile women (1997-2018) | | | | | |
| --- | --- | --- | --- | --- | --- |
|  | Population | |  | Cases | |
|  | No. | % |  | No. | % |
| **Age groups (years)** |  |  |  |  |  |
| 15**–**24 | 286,170 | 26.7 |  | 113,510 | 34.2 |
| 25**–**34 | 397,400 | 37.0 |  | 115,343 | 34.7 |
| 35**–**44 | 289,456 | 27.0 |  | 77,998 | 23.5 |
| 45**–**50 | 100,441 | 9.4 |  | 25,435 | 7.7 |
| **Educational level (years)** |  |  |  |  |  |
| ≤ 9 | 144,358 | 13.4 |  | 54,054 | 16.3 |
| 10**–**11 | 201,034 | 18.7 |  | 65,735 | 19.8 |
| ≥ 12 | 728,075 | 67.8 |  | 212,497 | 64.0 |
| **Family income (quartile)** |  |  |  |  |  |
| Low | 268,087 | 25.0 |  | 92,708 | 27.9 |
| Middle-low | 268,550 | 25.0 |  | 87,259 | 26.3 |
| Middle-high | 268,314 | 25.0 |  | 81,638 | 24.6 |
| High | 268,516 | 25.0 |  | 70,681 | 21.3 |
| **Region of residence** |  |  |  |  |  |
| Large cities | 671,418 | 62.5 |  | 224,061 | 67.4 |
| Southern Sweden | 282,866 | 26.4 |  | 78,201 | 23.5 |
| Northern Sweden | 119,183 | 11.1 |  | 30,024 | 9.0 |
| **Country of origin** |  |  |  |  |  |
| Sweden | 859,719 | 80.1 |  | 265,095 | 79.8 |
| Eastern Europe | 51,909 | 4.8 |  | 15,517 | 4.7 |
| Western countries | 35,536 | 3.3 |  | 9,747 | 2.9 |
| Middle East/North Africa | 58,205 | 5.4 |  | 20,967 | 6.3 |
| Africa (excluding North Africa) | 19,259 | 1.8 |  | 6,068 | 1.8 |
| Asia (excluding Middle East) and Oceania | 35,761 | 3.3 |  | 10,009 | 3.0 |
| Latin America and the Caribbean | 13,078 | 1.2 |  | 4,883 | 1.5 |
| **Body mass index (BMI)** |  |  |  |  |  |
| < 18.5 | 39,450 | 3.7 |  | 13,467 | 4.1 |
| 18.5**–**24.9 | 711,712 | 66.3 |  | 221,256 | 66.6 |
| 25.0**–**29.9 | 229,815 | 21.4 |  | 69,296 | 20.9 |
| ≥ 30.0 | 92,490 | 8.6 |  | 28,267 | 8.5 |
| **Body height (cm)** |  |  |  |  |  |
| < 161 | 206,710 | 19.3 |  | 64,372 | 19.4 |
| 161**–**170 | 603,086 | 56.2 |  | 185,380 | 55.8 |
| 171**–**180 | 252,998 | 23.6 |  | 79,105 | 23.8 |
| > 180 | 10,673 | 1.0 |  | 3,429 | 1.0 |
| **Parity** |  |  |  |  |  |
| 1 | 290,901 | 27.1 |  | 89,811 | 27.0 |
| 2 | 530,901 | 49.5 |  | 161,471 | 48.6 |
| ≥ 3 | 251,665 | 23.4 |  | 81,004 | 24.4 |
|  |  |  |  |  |  |
| **All** | **1,073,467** | **100.0** |  | **332 286** | **100.0** |

| **Supplementary Table S2.** Incidence rate (per 100 person-years) of cystitis in fertile women (1997-2018) | | | | | | |
| --- | --- | --- | --- | --- | --- | --- |
|  | IR | 95% CI | |  |  |  |
| **Age groups (years)** |  |  |  |  |  |  |
| 15**–**24 | 4.13 | 4.11 | 4.16 |  |  |  |
| 25**–**34 | 2.95 | 2.93 | 2.97 |  |  |  |
| 35**–**44 | 2.73 | 2.72 | 2.75 |  |  |  |
| 45**–**50 | 2.90 | 2.86 | 2.93 |  |  |  |
| **Educational level (years)** |  |  |  |  |  |  |
| ≤ 9 | 4.04 | 4.01 | 4.08 |  |  |  |
| 10**–**11 | 3.18 | 3.16 | 3.21 |  |  |  |
| ≥ 12 | 3.04 | 3.03 | 3.06 |  |  |  |
| **Family income** |  |  |  |  |  |  |
| Low | 3.67 | 3.65 | 3.69 |  |  |  |
| Middle-low | 3.15 | 3.13 | 3.17 |  |  |  |
| Middle-high | 3.06 | 3.04 | 3.08 |  |  |  |
| High | 2.92 | 2.90 | 2.94 |  |  |  |
| **Region of residence** |  |  |  |  |  |  |
| Large cities | 3.30 | 3.28 | 3.31 |  |  |  |
| Southern Sweden | 3.23 | 3.21 | 3.25 |  |  |  |
| Northern Sweden | 2.57 | 2.54 | 2.60 |  |  |  |
| **Country of origin** |  |  |  |  |  |  |
| Sweden (born in) | 3.07 | 3.06 | 3.09 |  |  |  |
| Eastern Europe | 3.55 | 3.50 | 3.61 |  |  |  |
| Western countries | 3.17 | 3.11 | 3.24 |  |  |  |
| Middle East/North Africa | 4.60 | 4.54 | 4.66 |  |  |  |
| Africa (excluding North Africa) | 4.23 | 4.12 | 4.33 |  |  |  |
| Asia (excluding Middle East) and Oceania | 3.31 | 3.24 | 3.37 |  |  |  |
| Latin America and the Caribbean | 4.23 | 4.11 | 4.35 |  |  |  |
| **Body mass index (BMI)** |  |  |  |  |  |  |
| < 18.5 | 3.61 | 3.55 | 3.68 |  |  |  |
| 18.5**–**24.9 | 3.19 | 3.18 | 3.21 |  |  |  |
| 25.0**–**29.9 | 3.14 | 3.12 | 3.16 |  |  |  |
| ≥ 30.0 | 3.23 | 3.19 | 3.26 |  |  |  |
| **Body Height (cm)** |  |  |  |  |  |  |
| < 161 | 3.34 | 3.32 | 3.37 |  |  |  |
| 161**–**170 | 3.16 | 3.14 | 3.17 |  |  |  |
| 171**–**180 | 3.18 | 3.16 | 3.20 |  |  |  |
| > 180 | 3.26 | 3.15 | 3.37 |  |  |  |
| **Parity** |  |  |  |  |  |  |
| 1 | 3.45 | 3.43 | 3.47 |  |  |  |
| 2 | 3.07 | 3.06 | 3.09 |  |  |  |
| ≥ 3 | 3.20 | 3.18 | 3.23 |  |  |  |
|  |  |  |  |  |  |  |
| **All** | **3.20** | **3.19** | **3.21** |  |  |  |

| **Supplementary Table S3.** Association between body height and cystitis in fertile women (adjusted for BMI, parity, and sociodemographic factors) | | | | | | | | | | | | | | | | | | | |
| --- | --- | --- | --- | --- | --- | --- | --- | --- | --- | --- | --- | --- | --- | --- | --- | --- | --- | --- | --- |
|  | Model 1 | | | |  | Model 2 | | | |  | Model 3 | | | |  | Model 4 | | | |
| Covariates | HR | 95% CI | | P-value |  | HR | 95% CI | | P-value |  | HR | 95% CI | | P-value |  | HR | 95% CI | | P-value |
| **Body height (ref. 161–170 cm)** |  |  |  |  |  |  |  |  |  |  |  |  |  |  |  |  |  |  |  |
| < 161 | 1.04 | 1.03 | 1.05 | <.0001 |  | 1.04 | 1.03 | 1.05 | <.0001 |  | 1.04 | 1.03 | 1.05 | <.0001 |  | 1.00 | 0.99 | 1.01 | 0.3201 |
| 171–180 | 1.01 | 1.01 | 1.02 | 0.0011 |  | 1.01 | 1.01 | 1.02 | 0.0024 |  | 1.01 | 1.00 | 1.02 | 0.0138 |  | 1.03 | 1.02 | 1.04 | <.0001 |
| > 180 | 1.04 | 1.01 | 1.08 | 0.0220 |  | 1.04 | 1.01 | 1.08 | 0.0220 |  | 1.03 | 0.99 | 1.06 | 0.1570 |  | 1.05 | 1.01 | 1.09 | 0.0055 |
| **Body mass index (BMI)** |  |  |  |  |  | 1.00 | 1.00 | 1.00 | <.0001 |  | 0.99 | 0.99 | 0.99 | <.0001 |  | 0.99 | 0.99 | 0.99 | <.0001 |
| **Parity** |  |  |  |  |  |  |  |  |  |  | 1.01 | 1.01 | 1.02 | <.0001 |  | 1.01 | 1.00 | 1.01 | 0.0004 |
| **Age (ref. age 45–50 years)** |  |  |  |  |  |  |  |  |  |  |  |  |  |  |  |  |  |  |  |
| 15**–**24 |  |  |  |  |  |  |  |  |  |  | 1.54 | 1.52 | 1.56 | <.0001 |  | 1.48 | 1.46 | 1.50 | <.0001 |
| 25**–**34 |  |  |  |  |  |  |  |  |  |  | 1.10 | 1.08 | 1.11 | <.0001 |  | 1.07 | 1.06 | 1.09 | <.0001 |
| 35**–**44 |  |  |  |  |  |  |  |  |  |  | 1.00 | 0.98 | 1.01 | 0.7727 |  | 0.97 | 0.96 | 0.99 | 0.0004 |
| **Educational level (ref. ≥ 12 years)** |  |  |  |  |  |  |  |  |  |  |  |  |  |  |  |  |  |  |  |
| ≤ 9 |  |  |  |  |  |  |  |  |  |  |  |  |  |  |  | 1.18 | 1.17 | 1.19 | <.0001 |
| 10**–**11 |  |  |  |  |  |  |  |  |  |  |  |  |  |  |  | 1.12 | 1.11 | 1.13 | <.0001 |
| **Family income (ref. High)** |  |  |  |  |  |  |  |  |  |  |  |  |  |  |  |  |  |  |  |
| Low |  |  |  |  |  |  |  |  |  |  |  |  |  |  |  | 1.07 | 1.06 | 1.08 | <.0001 |
| Middle low |  |  |  |  |  |  |  |  |  |  |  |  |  |  |  | 1.05 | 1.04 | 1.06 | <.0001 |
| Middle high |  |  |  |  |  |  |  |  |  |  |  |  |  |  |  | 1.03 | 1.02 | 1.04 | <.0001 |
| **Region of residence (ref. Large cities)** |  |  |  |  |  |  |  |  |  |  |  |  |  |  |  |  |  |  |  |
| Southern Sweden |  |  |  |  |  |  |  |  |  |  |  |  |  |  |  | 0.93 | 0.92 | 0.94 | <.0001 |
| Northern Sweden |  |  |  |  |  |  |  |  |  |  |  |  |  |  |  | 0.77 | 0.76 | 0.78 | <.0001 |
| **Country of origin (ref. Born in Sweden)** |  |  |  |  |  |  |  |  |  |  |  |  |  |  |  |  |  |  |  |
| Eastern Europe |  |  |  |  |  |  |  |  |  |  |  |  |  |  |  | 1.03 | 1.02 | 1.05 | <.0001 |
| Western countries |  |  |  |  |  |  |  |  |  |  |  |  |  |  |  | 1.04 | 1.02 | 1.06 | 0.0001 |
| Middle East/North Africa |  |  |  |  |  |  |  |  |  |  |  |  |  |  |  | 1.27 | 1.25 | 1.29 | <.0001 |
| Africa (excluding North Africa) |  |  |  |  |  |  |  |  |  |  |  |  |  |  |  | 1.12 | 1.09 | 1.15 | <.0001 |
| Asia (excluding Middle East) and Oceania |  |  |  |  |  |  |  |  |  |  |  |  |  |  |  | 0.97 | 0.95 | 0.99 | 0.0016 |
| Latin America and the Caribbean |  |  |  |  |  |  |  |  |  |  |  |  |  |  |  | 1.29 | 1.25 | 1.32 | <.0001 |
| Model 1: Univariate model; Model 2: Adjusted for BMI; Model 3: Adjusted for BMI, age, and parity; Model 4. Fully adjusted. | | | | | | | | | | | | | | | | | | | |

| **Supplementary Table S4.** Association between individual characteristics and uncomplicated cystitis in women aged 15-50 years (univariate model) | | | | | |
| --- | --- | --- | --- | --- | --- |
| Covariates | HR | 95% CI | | P-value |  |
| **Body mass index (BMI) (ref. 18.5-24.9)** |  |  |  |  |  |
| <18.5 | 1.12 | 1.10 | 1.14 | <.0001 |  |
| 25.0-29.9 | 0.98 | 0.97 | 0.99 | <.0001 |  |
| ≥ 30.0 | 1.00 | 0.98 | 1.01 | 0.6069 |  |
| **Body height (cm) (ref. 161-170)** |  |  |  |  |  |
| <161 | 1.04 | 1.03 | 1.05 | <.0001 |  |
| 171-180 | 1.01 | 1.01 | 1.02 | 0.0011 |  |
| >180 | 1.04 | 1.01 | 1.08 | 0.0220 |  |
| **Parities** | 1.00 | 0.99 | 1.00 | 0.0069 |  |
| **Age (ref. age 45-50 years)** |  |  |  |  |  |
| 15-24 | 1.51 | 1.49 | 1.53 | <.0001 |  |
| 25-34 | 1.08 | 1.07 | 1.10 | <.0001 |  |
| 35-44 | 0.99 | 0.98 | 1.01 | 0.2064 |  |
| **Educational level (ref. ≥ 12 years)** |  |  |  |  |  |
| ≤ 9 | 1.32 | 1.31 | 1.34 | <.0001 |  |
| 10-11 | 1.06 | 1.05 | 1.07 | <.0001 |  |
| **Family income (ref. High)** |  |  |  |  |  |
| Low | 1.29 | 1.28 | 1.30 | <.0001 |  |
| Middle low | 1.13 | 1.12 | 1.14 | <.0001 |  |
| Middle high | 1.08 | 1.07 | 1.09 | <.0001 |  |
| **Region of residence (ref. Large cities)** |  |  |  |  |  |
| Southern Sweden | 0.92 | 0.91 | 0.93 | <.0001 |  |
| Northern Sweden | 0.77 | 0.76 | 0.78 | <.0001 |  |
| **Country of origin (ref. Born in Sweden)** |  |  |  |  |  |
| Eastern Europe | 1.08 | 1.06 | 1.09 | <.0001 |  |
| Western countries | 0.98 | 0.96 | 1.00 | 0.1025 |  |
| Middle East/North Africa | 1.37 | 1.35 | 1.39 | <.0001 |  |
| Africa (excluding North Africa) | 1.23 | 1.20 | 1.26 | <.0001 |  |
| Asia (excluding Middle East) and Oceania | 1.00 | 0.98 | 1.02 | 0.7154 |  |
| Latin America and the Caribbean | 1.31 | 1.27 | 1.35 | <.0001 |  |

**Supplementary Figure S1**. Incidence rate (per 100 person-years) of cystitis in fertile women by BMI and Country of origin (immigration status)


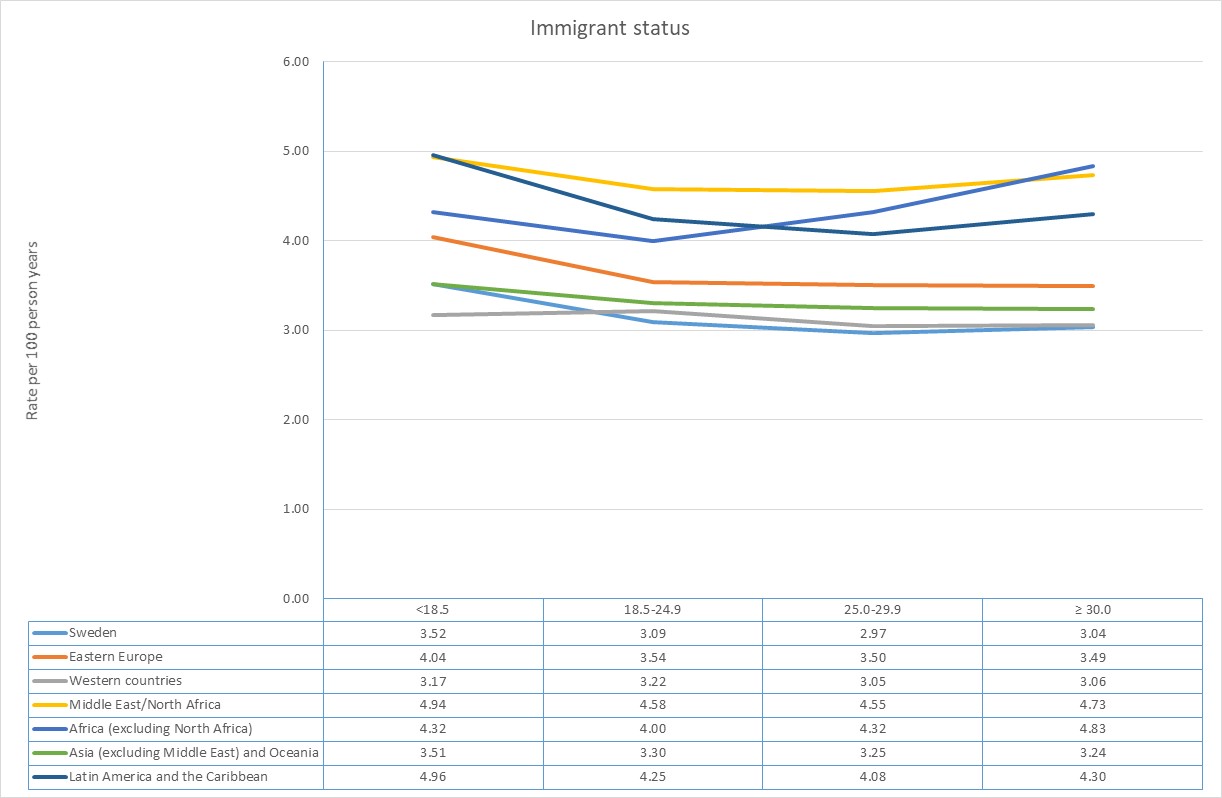


**Supplementary Figure S2**. Incidence rate (per 100 person-years) of cystitis in fertile women by BMI and Age**
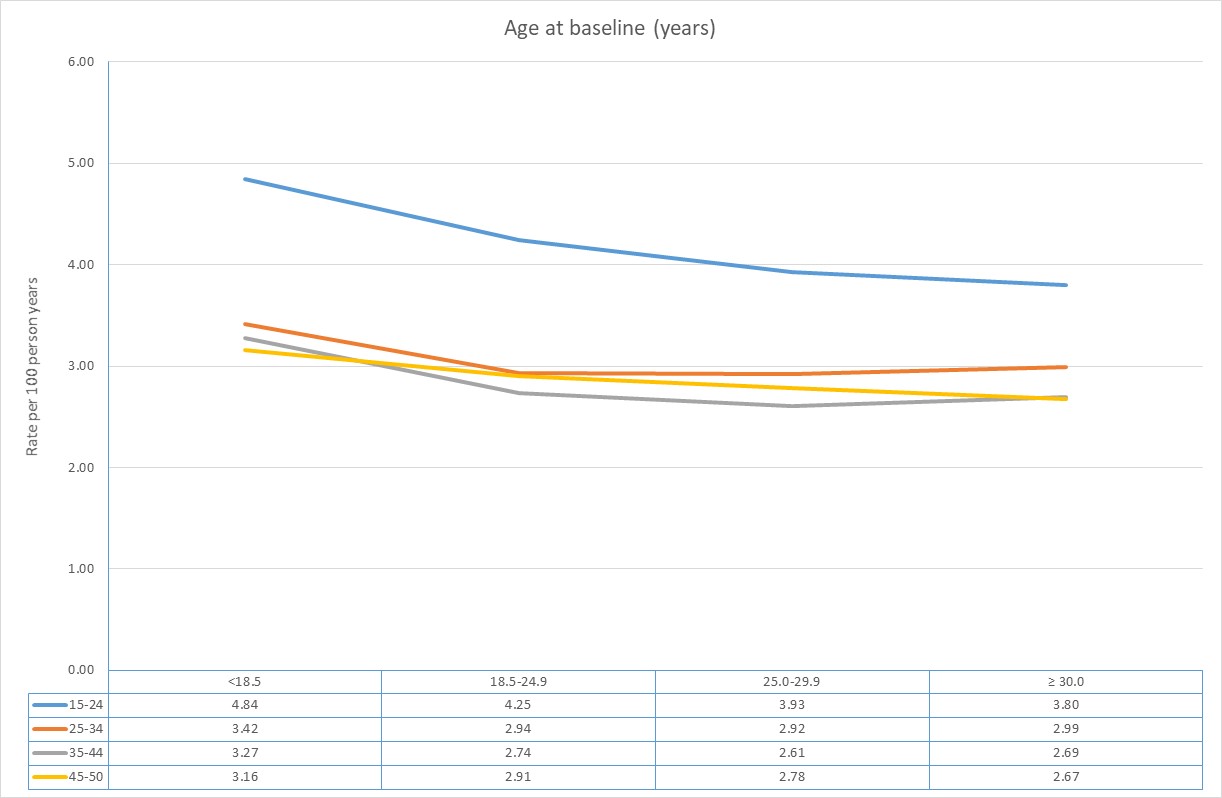
**
